# Acompaniment in Grief. Times of Coronavirus

**DOI:** 10.3389/fpsyg.2020.583233

**Published:** 2020-10-15

**Authors:** Jose Carlos Bermejo

**Affiliations:** Centro de Humanización de la Salud, Centro San Camilo, Madrid, Spain

**Keywords:** bereavement, pandemic (COVID-19), grief source: DeCS, process of dying, dignified death

Reflection on grief, the loss of a loved one, and the way of accompanying those who experience this suffering, is quite scarce. In some way, grief constitutes one of those taboo topics with which we are not educated to live healthily either, but it is the force of the experience of death that gives us some key to face it. Nowadays, there is also a need for reflecting about how to support mourning and grief of those people who experience the loss of loved ones, without even having the chance to properly celebrate and accompany their dying, due to stringent Coronavirus measures of social distancing and thereby prevention of contagion.

Given the cultural resistance, abandonment, and active denial of death and dying and seeking full life, this article addresses a truly relevant and necessary discussion about the meaning of death. With the experience of the Coronavirus, the literature on support for death has grown. Even though there are few research studies focused on outcomes and support for bereaved people during a pandemic (Mayland et al., 2020). Studies tend to focus on survivors (Carr et al., 2020).

As with previous pandemics, through this one, multiple losses have been caused, related to death itself and also to disruption in rituals and mourning practices, and therefore increasing the risk of complicated grief (Mayland et al., 2020). As in other studies (LeRoy et al., 2020), we try to describe how to help (sick and terminally ill, dying) to cope with proximity and the experience of dying, how to help family members/loved ones/caregivers, how psychologists and health professionals can help, and whether this should be done in general or, in particular, during these times of the coronavirus.

The topic, however, is neglected in modern medicine and in the therapeutic fields, which has been widely acknowledged throughout history. In fact, medieval treatises began death education and have explored it at different depths in philosophy, psychology, anthropology (Thomas, 2019). Thanatology is a recently emerging field that contemplates death studies. It is a complex and multidisciplinary field that encompasses the range of human experiences, emotions, expectations, and realities (Becker, 1973; Meagher and Balk, 2013). In fact, there has always been a need of finding meaning, giving continuity, or transcending death (Kübler-Ross, 1969, 2005; Lifton, 1979).

The science of death and dying emerged in a historical context marked by intense social, economic, and political changes that in turn has contributed to the concept of death being excluded from social life (Fonseca and Testoni, 2011). On the occasion of the coronavirus pandemic, society has become more aware of the value of accompaniment at the end of life and in mourning. The legal ban on visiting hospitals (except for small meetings in terminal situations), as well as participating in grief, both in morgues and rites, has put on the table a reality already existing before, in other contexts. Certainly, some immigrants have encountered in this way the end and mourning of their loved ones; some did not even have any information about their deaths. However, on the occasion of the pandemic, this situation has been escalated and universalized for months.

Is it possible to think that the death of the others, besides tearing, can teach us to live and humanize? Is there anything positive that we can find in death or in the process of dying when they have been deprived of their community dimension, of the accompaniment of those who lived their own death, or that of loved ones separated from their families? Can this contribute something to being happier? Isn't the death of a loved one a painful loss that, above all, tears us apart and puts us in crisis? Does psychological intervention help and prevent something?

Dying and death demand truth, and we learn certain truths that can contribute to humanizing us (Bermejo, 2009a).

Death puts us hopelessly in front of the mystery of life. It imposes silence and inevitable reflection on us. And, in a way, it makes us all philosophers, thinkers about the ultimate meaning of life, relationships, and love. Death does not trigger any thought, but intensely felt thoughts. It is like living before an enigma that possesses us, as the pregnant woman possesses her child to give meaning. Death, thus, can teach us to live and humanize and accompany these processes from psychology. It represents an ethical duty and an opportunity to generate biographical health.

Accompanying in dying, also by telephone and video call, can teach us to live because it claims values that can easily be relegated in daily life, values evoked more by feeling than by reason, values that claim relationship and accompaniment.

This topic “not knowing what to say,” which is typical of accompaniment at the end of life and in mourning, is as significant because it reveals our identities of limitation, vulnerability, and poverty. It reveals the value of our silent and absent presence, the value of the embrace, which cannot now be given, and of the hand stretched—in the metaphorical sense—of the caress now impossible (Bermejo, 2020a); it reveals the power of the small and simple, the need for the symbolic to survive, to continue living.

Dying according to these parameters is a feature to speak of a dignified death.

Human beings desire the possibility of living their own deaths, not to be deceased by others. Not being able to accompany loved ones due to the coronavirus pandemic situation has generated a certain expropriation of this possibility. This is the first characteristic that a dignified death should have: an appropriate death, not expropriated, as Tolstoy so clearly makes us see in *The Death of Ivan Illich* (Illich, 1997). That chain of calls and messages in which the patient is spoken, but without the patient, generates a very notable isolation in the patient.

Dying worthily (Küng and Walter, 1997) consists of making the effort to characterize the personal process and accompanying from the environment to make a final narrative. Each person, thus, could imagine his own process, describing it with proper personal qualifiers, which make this moment of his existence such an important moment that it is, definitely, the last. Hardly a person would wish it in the loneliness and impossibility of face-to-face communication.

A death will be more dignified the more it is spoken by the subject and the people who are most affected. A spoken death is one in which there is room for the voice, for the words around death, where you can hear what is said and what is not said.

A dignified death will be one that deserves the adjective of beautiful, but not in an idealized sense, but a death in which the person lives until the last moment. The isolation by coronavirus has generated outrage due to the feeling that my beloved one is not dying close to me but among others, among other sick persons and professionals, who have been entrusted with a strange accompaniment.

A characterized death (Bermejo, 2009b) is the one in which the subjects are happy to live, that is, saved by death, because to kill death would be to feel how love and solidarity dies. It is death that gives ultimate meaning to our lives. It will be satisfied if we are able to fill our relationships with content (*con-tenti*) and communion. Characterizing death entails doing what we can for constructing the process of dying as a dimension of life, in other words, learning to lose and to progressively integrate our condition of finesse (Bermejo and Magaña, 2014).

Talking about dignified death means working toward the person governing himself in the best of his means, thus ruling the space (physical, personal, affective, etc.) that surrounds him in the last months or days, to the extent that nature and personal limitation permits. The coronavirus has pushed the vital world out of the patient, creating suffering added to the process of dying and losing.

A humanized death is one where the legitimate rarity of each one can be developed, where the feelings, desires, desired companies or not, and expectations can be adequately expressed…

A dignified death would be one that becomes a true experience of love because the death experience is made only by the one who loves. We should talk about death as lovers speak, who love life because it is limited, because they want to make the most of life and joy at every moment.

Death should be an exercise in learning, of art, because the only thing is the “*ars vivendi*” and the “*ars moriendi*” when the idea that dying is an instant and conceived as a process in the human journey toward the realization of who we are and what we are called to be has been overcome. Those who have been sick with coronavirus and family members have been forced to learn an “*ars novus*” (Paglia, 2017).

It is more human the death that can be narrated. Because, deep down, we cannot talk about that; the best thing that can be done is to narrate it. A narrated death allows it to be characterized by oneself, by loved ones. It finds no explanation for reasons but fills it with words when it talks about how.

Ultimately, a healthy characterized death can be called an elegant death because it is tailored to the responsible, the capacity for one's own personal choice (election), considering life as a gift for those who interpret it, and it does not lead us to live exclusively to gambling of the capricious non-rational nature (Requena, 2017).

The coronavirus has made dying an archipelago, which is characterized precisely of being united by what it separates.

Adjectivized death would thus become a mystery experience rather than a simple problem to manage. Mystery is not something that is outside of us and has a solution. The mystery is within us; it surrounds us, and we have no choice but to live with it. Living it humanly entails the ultimate expression of the health of a person, which results in “*meditatio mortis*” that will not be the unpleasant obsession but the human understanding of the ultimate value of life in view of its end, in the midst of the dynamism of hope (Bermejo, 2020b).

Health professionals, sufficiently trained in psychology, particularly in counseling, will help prevent complicated and pathological grief if they accompany healthy processes at the end of the lives of loved ones, playing special mediators, sent in times of pandemic, transmitting messages, reading letters, interpreting, etc. to encourage possible communication.

## Author Contributions

The whole article has been written by JB.

## Conflict of Interest

The author declares that the research was conducted in the absence of any commercial or financial relationships that could be construed as a potential conflict of interest.
